# Multi-trait QTL analysis for agronomic and quality characters of *Agaricus bisporus* (button mushrooms)

**DOI:** 10.1186/s13568-016-0239-3

**Published:** 2016-09-08

**Authors:** Wei Gao, Johan J. P. Baars, Chris Maliepaard, Richard G. F. Visser, Jinxia Zhang, Anton S. M. Sonnenberg

**Affiliations:** 1 Wageningen UR Plant Breeding, Wageningen University & Research Centre, Droevendaalsestaag 1, 6700 AA Wageningen, The Netherlands; 2 Institute of Agricultural Resources and Regional Planning, Chinese Academy of Agricultural Sciences, Zhongguancun South Street 12, Beijing, 100081 People’s Republic of China

**Keywords:** Multi-trait QTL analysis, Genetic correlations, Phenotypic correlations, QTLs on the same chromosome

## Abstract

The demand for button mushrooms of high quality is increasing. Superior button mushroom varieties require the combination of multiple traits to maximize productivity and quality. Very often these traits are correlated and should, therefore, be evaluated together rather than as single traits. In order to unravel the genetic architecture of multiple traits of *Agaricus bisporus* and the genetic correlations among traits, we have investigated a total of six agronomic and quality traits through multi-trait QTL analyses in a mixed-model. Traits were evaluated in three heterokaryon sets. Significant phenotypic correlations were observed among traits. For instance, earliness (ER) correlated to firmness (FM), cap color, and compost colonization, and FM correlated to scales (SC). QTLs of different traits located on the same chromosomes genetically explains the phenotypic correlations. QTL detected on chromosome 10 mainly affects three traits, i.e., ER, FM and SC. It explained 31.4 % phenotypic variation of SC on mushroom cap (heterokaryon Set 1), 14.9 % that of the FM (heterokaryon Set 3), and 14.2 % that of ER (heterokaryon Set 3). High value alleles from the wild parental line showed beneficial effects for several traits, suggesting that the wild germplasm is a valuable donor in terms of those traits. Due to the limitations of recombination pattern, we only made a start at understanding the genetic base for several agronomic and quality traits in button mushrooms.

**Electronic supplementary material:**

The online version of this article (doi:10.1186/s13568-016-0239-3) contains supplementary material, which is available to authorized users.

## Introduction

Button mushrooms are cultivated worldwide with a product volume of 3.9 million tonnes and a total value of 4.7 billion dollars in 2009, mainly produced in China, the USA, Poland, the Netherlands, South Korea and France (Sonnenberg et al. 2011). During the last 30 years, mushroom productivity has increased substantially as a result of improved composting techniques and optimized environmental conditions, and to a lesser extent by breeding efforts. The first hybrid cultivar Horst U1 (Fritsche 1981), was released in the 1980s. Subsequent new varieties were identical or very similar to this first hybrid indicating that these were all varieties derived from the first hybrid line (Sonnenberg et al. 2005, 2016). Mushroom breeding has been so far an applied science and new varieties have been generated mainly modifying existing varieties by selecting fertile single spore cultures or by generating multi spore cultures. Generating new varieties with sufficient qualities by outbreeding appears to be a difficult task (personal communications with spawn companies and personal experiences). No elite stocks are available for mushroom breeders, which means that for the introduction of new traits wild germplasm has to be used. Since knowledge on the genetic base of most traits is unknown, the use of wild lines leads to a long breeding program with uncertain outcomes.

The Homobasidiomycetes, among which *Agaricus bisporus* (button mushroom), are characterized by the fact that they contain two types of haploid nuclei with different mating types that remain side by side in each cell. Fusion of nuclei only takes place in basidial cells just before meiosis. Most basidia of *A. bisporus* produce only two spores and the four post-meiotic nuclei are distributed over two spores in such a way that non-sister nuclei are paired in one spore (Elliott 1972; Summerbell et al. 1989). This usually leads to mycelia with two different mating types and thus to fertile heterokaryons. This type of life cycle is designated as secondary homothallic. This phenomenon is also referred to as automixis or intra-tetrad mating, a form of selfing where mating occurs among the products of a single meiosis. In rare cases, basidia produce three or four spores. Only on these basidia spores are produced with one haploid nucleus that generate homokaryons and can be used for cross breeding. Two decades ago, a novel variety has been found in de Sonoran desert of California (Callac et al. 1993). This variety produces predominantly four-spored basidia and each spore germinates into homokaryotic mycelia. The two varieties are designated as *A. bisporus* var. *bisporus* and *A. bisporus* var. *burnettii*, respectively. Next to differences in average spore number per basidium, both species also differ in recombination landscape. Offspring of *A. bisporus* var. *bisporus* show crossover (CO) mainly restricted to regions ca 100 kb from chromosome ends (Sonnenberg et al. 2016). Offspring of hybrids between the *A. bisporus* var. *bisporus* and var. *burnettii* show COs distributed over the whole chromosome indicating that the var. *burnettii* has a more evenly distribution of CO (Foulongne-Oriol et al. 2010). The typical restricted CO landscape of the var. *bisporus* results in a fact that the major part of chromosomes of the offspring are parental types. Quantitative trait locus (QTL) mapping for this variety is thus mainly restricted to assignment of QTL to chromosomes (Gao et al. 2015). Introduction of new traits in the *bisporus* variety is thus problematic due to large linkage drag. Most strains of the var. *burnettii* are poor in various agronomic traits and have not been used up till now to generate new cultivars.

As in plants, fungal lines with contrasting performance are commonly crossed to generate segregating populations and used to map genomic regions involved in traits and to identify candidate genes (Foulongne-Oriol 2012). This is usually done by protoplasting the heterokaryotic parental lines and recovering the constituent nuclei as homokaryons which are subsequently used for outcrossing. In the button mushroom, mating between homokaryons is controlled by one mating type locus (MTA) (Xu et al. 1993). Homokaryotic offspring can be used directly as a segregating population for genetic linkage mapping (haplotyping). Since homokaryons are infertile they have to be crossed with a compatible homokaryotic tester line to produce mushrooms for QTL mapping. Genetic linkage maps have been generated so far in both bisporic populations of var. *bisporus* and intervarietal populations (Foulongne-Oriol et al. 2010; Gao et al. 2015; Kerrigan et al. 1993). The- haploid genome size of *A. bisporus* is 30.4 Mb, it contains 13 chromosomes and has been sequenced completely (Morin et al. 2012).

New button mushroom varieties must at least meet the standards of the present varieties in order to be commercially viable. Important agronomic and quality traits are yield, earliness (ER) (first harvesting day), maturation (cap opening during mushroom development), size, color, shape, firmness (FM), aroma, scaling (smoothness of the cap skin) and shelf life (post-harvest discoloration) (Gao et al. 2015; Pardo et al. 2010). Most of these traits are quantitative and have a complex genetic basis, associated with quantitative trait loci (QTLs). In *A. bisporus* only a few genetic studies have been conducted to unravel the mechanisms of the agronomic and quality traits mentioned above (Callac et al. 1998; Foulongne-Oriol et al. 2011, 2012a, b). Although some of these QTL studies considered multiple traits, they were analysed separately thus neglecting possible genetic correlations between traits. Agronomic traits and quality traits are often interrelated. For instance, a larger number of mushrooms correlated with a greater overall yield (a total of three or four flushes), a smaller cap size, and an earlier first flush (Larraya et al. 2002, 2003). Similarly, a positive correlation was observed between ER and yield, and the earliest genotype tended to have higher yield and produce a larger number of smaller mushrooms (Foulongne-Oriol et al. 2012a). An integrated analysis by combining multiple traits can be more powerful than a collection of single-trait analyses and allows a more realistic analysis of the data since genetic correlations between traits can be directly modelled (Malosetti et al. 2008). Furthermore, the models of multi-trait QTL analysis allow the detection of closely linked QTLs or pleiotropic chromosomal regions that result in genetic correlations among traits (van Eeuwijk et al. 2010). The aim of multi-trait QTL analysis is to describe the genetic variation within traits, and the genetic correlations among traits in terms of the signs and magnitudes of QTL effects.

In this study, a total of six agronomic and quality traits of button mushroom were recorded in two segregating populations during the spawn-run and fruiting period. The aim of this study was to identify the genetic basis of the overall quality of button mushroom with multi-trait QTL analyses and to dissect the genetic correlations among these traits. To our knowledge, this is the first multi-trait QTL analysis for mushroom quality.

## Materials and methods

### Lines and segregating populations

**Fig. 1:**
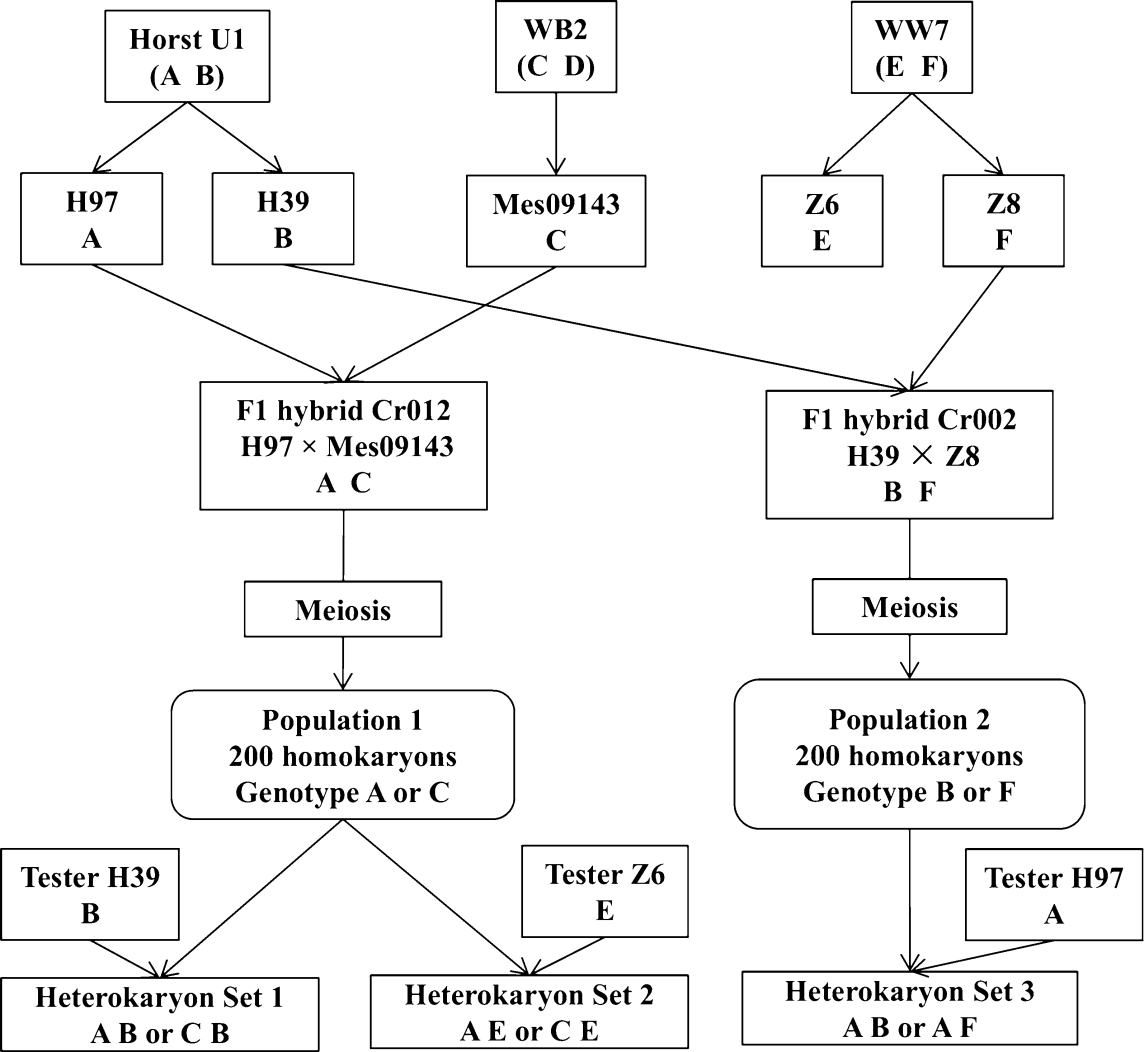
Flow diagram to illustrate the generation of the three segregating populations used in this study. Capital letters “A to F” represents the genotypes of the parental lines. Adapted from Gao et al. (2015)

Two sets of 200 homokaryotic progeny and three sets of heterokaryons of *A. bisporus* var. *bisporus* were developed in a previous study directed to detect QTL for cap discoloration after mechanical damage, i.e. bruising sensitivity (Gao et al. 2015). These lines also showed a substantial variation in agronomic and quality traits, and they were thus used in a separate analysis reported here to study segregation for those traits. Briefly, Population 1 represents the offspring of a parental homokaryon of Horst U1 (H97) and a parental homokaryon of a wild line (Mes09143). The individuals were either crossed with tester line H39 (the other parental homokaryon of Horst U1) or with tester line Z6 (a parental homokaryon of wild line WW7). In this way we could study the segregation in a mainly Horst U1 background or a mainly wild (WW7) background (Fig. 1). Horst U1 represents the present standard for agronomic and quality traits for button mushroom since most present-day commercial white varieties are derived from this hybrid (Sonnenberg et al. 2016). In this way we can see how the introduction of wild germplasm affects its important traits. Population 2 represents the offspring of a parental homokaryon of Horst U1 (H39) and a parental homokaryon of a wild line (Z8). These individuals were crossed with tester line H97 (the other parental homokaryon of Horst U1). This allows the study of another wild germplasm in the genetic background of Horst U1. The three heterokaryotic parental lines (Horst U1, WB2 and WW7), and the two F1 hybrids (Cr012 and Cr002) were also included in the cultivation trials for phenotyping (Gao et al. 2015). A total of 191 individuals (hybrids) of heterokaryon Set 1 (Population 1 crossed with tester line H39), 185 individuals of Set 2 (Population 1 crossed with tester line Z6), and 180 individuals of Set 3 (Population 2 crossed with tester line H97) produced enough mushrooms for phenotypic evaluation. The parental lines and all homokaryons of the two populations used in this study were deposited and publicly available in the fungal collection of Plant Breeding Wageningen UR (Additional file 1: Table S1).

### Phenotypic evaluation

Independent cultivation trials were carried out successively for each of the three heterokaryon sets at the mushroom farm of Unifarm in Wageningen UR with controlled climate. Mushrooms were grown as described previously (Gao et al. 2015).

The level of compost colonization (COCO) per genotype was recorded on a scale of 1–10 on day 14 after spawning (Table 1), where a score 1 indicates very poorly colonized compost (or 10 % surface colonization), and score 10 indicates very good colonization (or 100 % surface colonization). All trays were covered with casing soil on the same day. ER was scored as time (days) from spawning (inoculation) until the first day of harvest at developmental stage 3 (Hammond and Nichols 1976), and a high value for ER indicates late production. The other four traits (cap color, FM, scaling, and distribution) were also scored at developmental stage 3. All traits were scored as an average of mushrooms of a whole tray. Cap color (CC) was scored as 1–5, and a high value indicates dark mushrooms. Firmness was estimated from 1 (very weak) to 5 (very firm) by squeezing the mushroom cap with thumb and index finger placed on opposite site of the cap. Scaling (SC) was also measured from 1 to 5, where 1 indicate heavy scaling and 5 indicates smooth mushroom caps. Some genotypes produce mushrooms as clusters on the growing beds that is an undesirable trait for growers. The production of all mushrooms as clusters was scored as “1”, and an even distribution of individual mushrooms was scored as “5”.

**Table 1:** Quality related traits observed in the three sets of heterokaryons

Abbreviations	Quality traits	Data scale	Definition
COCO	Compost colonization	1–10	1 = very bad; 10 = very good
ER	Earliness		Time (days) from spawning to first harvest day ranging from 35 to 55 days
CC	Cap colour	1–5	1 = white, 2 = off-white, 3 = light brown, 4 = brown, 5 = dark brown
FM	Firmness of caps	1–5	1 = very weak; 5 = very firm
SC	Scales on caps	1–5	1 = strongly scaled; 5 = smooth
DS	Mushroom distribution	1–5	1 = clustered; 5 = even

### Statistical analysis

All statistical analyses were performed with the program Genstat, version 15. Since five lines including the three heterokaryotic parental lines (Horst U1, WB2, WW7) and two F1 hybrids (Cr012, Cr002) were grown three times as control lines in three cultivation tests (Gao et al. 2015), their performances for all traits were calculated as the mean values over the three cultivation tests. Lines were considered to be different if the mean values differed by more than the least significant difference (LSD) at α = 0.05. Data of heterokaryon Set 1 and Set 2 were used to assess the significance of the tester effect. Broad-sense heritability (*H*^2^) was calculated across tester lines (over heterokaryon Set 1 and Set 2) as follows: $H^{2} = \sigma_{G}^{2} / [\sigma_{G}^{2} + (\sigma_{e}^{2} /n)]$, where $\sigma_{G}^{2}$ represents the genotypic variance, $\sigma_{e}^{2}$ represents the residual variance, and n is the number of testers (indicate heterokaryon set for which this was the case) (n = 2). Trait correlations were calculated using Spearman’s rank correlation coefficient.

### Genetic linkage map

The segregation analysis and genetic linkage maps of the two homokaryotic populations were reported in a previous study (Gao et al. 2015). Briefly, the total map length of the first homokaryotic population (heterokaryon Set 1 and Set 2 in this study) was 164 cM generated with 95 SNP markers selected according to the genome sequence (13 chromosomes, 30.4 Mb in total). It has an average crossover frequency of 0.1 per individual per chromosome. The map of the second homokaryotic population (heterokaryon Set 3 in this study) was 86 cM generated with 76 SNP markers, and it has an average crossover frequency of 0.05 per individual per chromosome. SNP markers used for genotyping were selected to make them evenly distribute based on the genome sequences of the homokaryon H97 (Morin et al. 2012). Nevertheless, the linkage maps are very short with regions of low resolution because of the low recombination frequency observed mainly due to restriction of CO at chromosome ends (Sonnenberg et al. 2016). The QTL mapping is thus restricted in most cases to assigning QTLs to chromosomes.

### QTL analysis

QTL analyses were conducted for each of the three sets of heterokaryons. Scores of traits were standardized by subtracting the mean and dividing by the standard deviation. Multi-trait QTL analysis was carried out with the statistical program Genstat, version 15. The variance–covariance model for QTL selection and fitting the QTL model was chosen as “unstructured”, based on the Bayesian information criterion (BIC) (Boer et al. 2007; Malosetti et al. 2008). The threshold −log_10_(*p* value) for QTL detection was calculated with a genome wide significance threshold of *p* < 0.05 (Boer et al. 2007; Li and Ji 2005). Simple interval mapping was used for an initial genome wide scan to select significant candidate QTLs based on the threshold [−log_10_(*p* value)], and the ones having the highest [−log_10_(*p* value)] were selected as cofactors, then several rounds of composite interval mapping (CIM) were done until no new QTLs were detected. A REML (residual maximum likelihood) procedure was used iteratively to fit the final QTL model at each linkage group position. The upper bound and lower bound of QTL positions (confidential interval) on each chromosome were also calculated with the threshold significance. Results of multi-trait analyses were compared to those of single trait analyses and verified.

## Results

### Performance of control lines

The mean performance of the control lines (parental lines and F1 hybrids) across the three cultivation tests was evaluated. The CC of Horst U1, WW7 and Cr002 were consistently scored as “1” since they were white; WB2 and Cr012 were consistently scored as “3” (light brown) in all three cultivation tests. Since none of these controls showed variation in CC, no statistical analysis was done. CC was not scored in heterokaryon Set 3 since both the parental homokaryons and the tester homokaryon are derived from white heterokaryons. The five control lines did not show significant differences in two traits: COCO, and DS (Table 2). The brown wild line WB2 produced significantly earlier than the white commercial hybrid Horst U1 by more than 2 days, but ER of Horst U1 was not significantly different from that of the white wild line WW7. The F1 hybrid Cr012 resembled its brown parental heterokaryon WB2, which also produced significantly earlier than Horst U1; F1 hybrid Cr002 did not show significant difference in ER compared to its two parental heterokaryons (Horst U1 and WW7). The original heterokaryons did not differ significantly in FM although Horst U1 seems to be a bit firmer than the others. WW7 and Cr002 were significantly smoother (higher SC) than the commercial line U1.

**Table 2:** Trait performance of heterokaryotic parental lines and F1 hybrids

Line	COCO	ER	DS	FM	SC
Horst U1	9.0	44.3^b^	3.3	4.3^b^	2.3^ab^
WB2	9.3	41.0^a^	2.0	3.3^ab^	3.3^bc^
WW7	9.7	43.0^ab^	2.3	3.7^ab^	4.0^c^
Cr012	10.0	41.0^a^	2.7	3.3^ab^	1.3^a^
Cr002	9.3	43.3^b^	1.3	2.7^a^	4.7^c^

Significant differences by multiple comparison (LSD) was indicated with superscript letters (*α* = 0.05)

### Statistics for three sets of heterokaryons

**Fig. 2:**
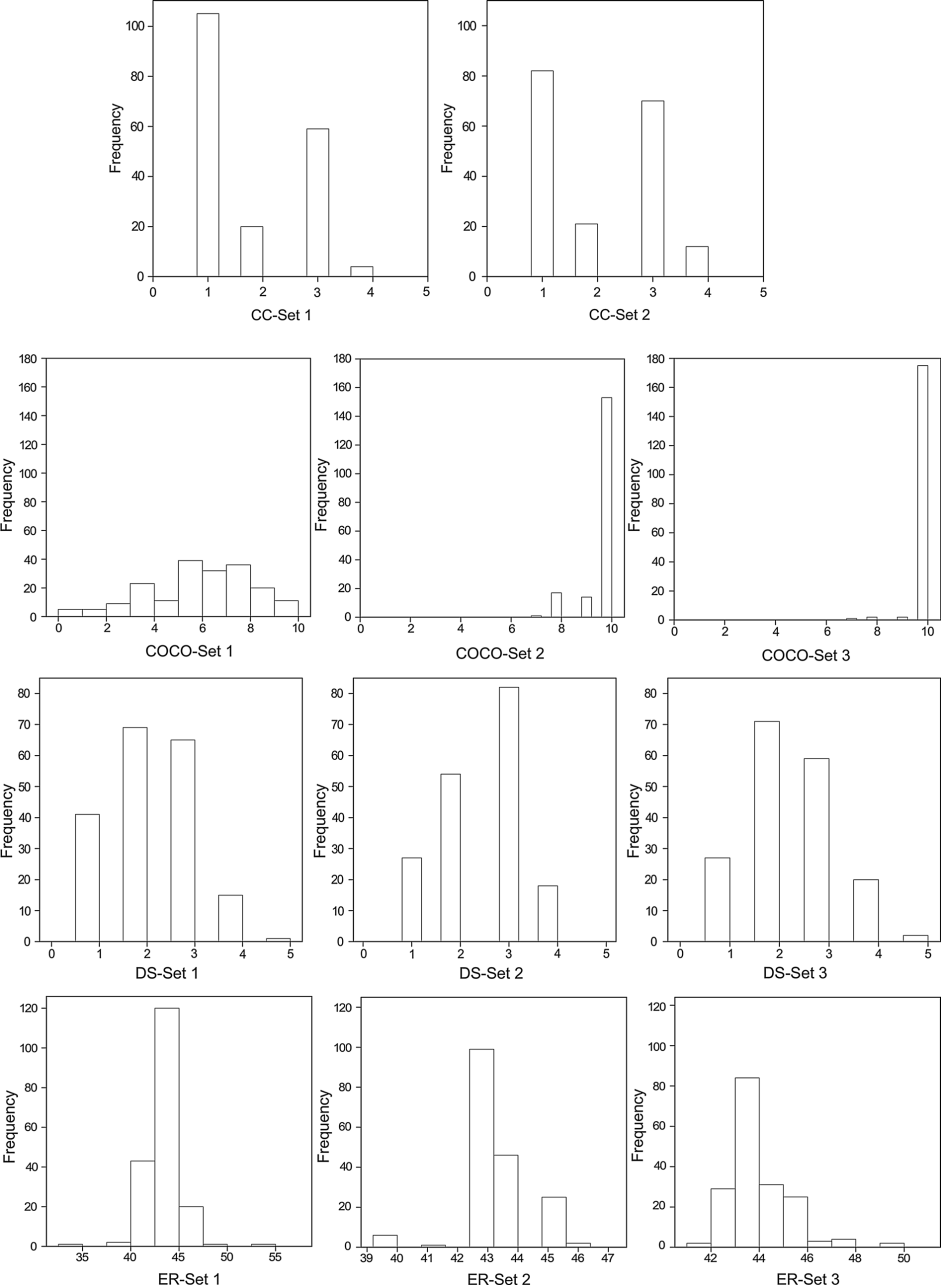
Frequency distribution histograms for traits CC, COCO, DS, and ER of heterokaryon Set 1, Set 2 and Set 3. The *horizontal axis* indicates data range of traits, and the *vertical axis* indicates the frequency of individuals

**Fig. 3:**
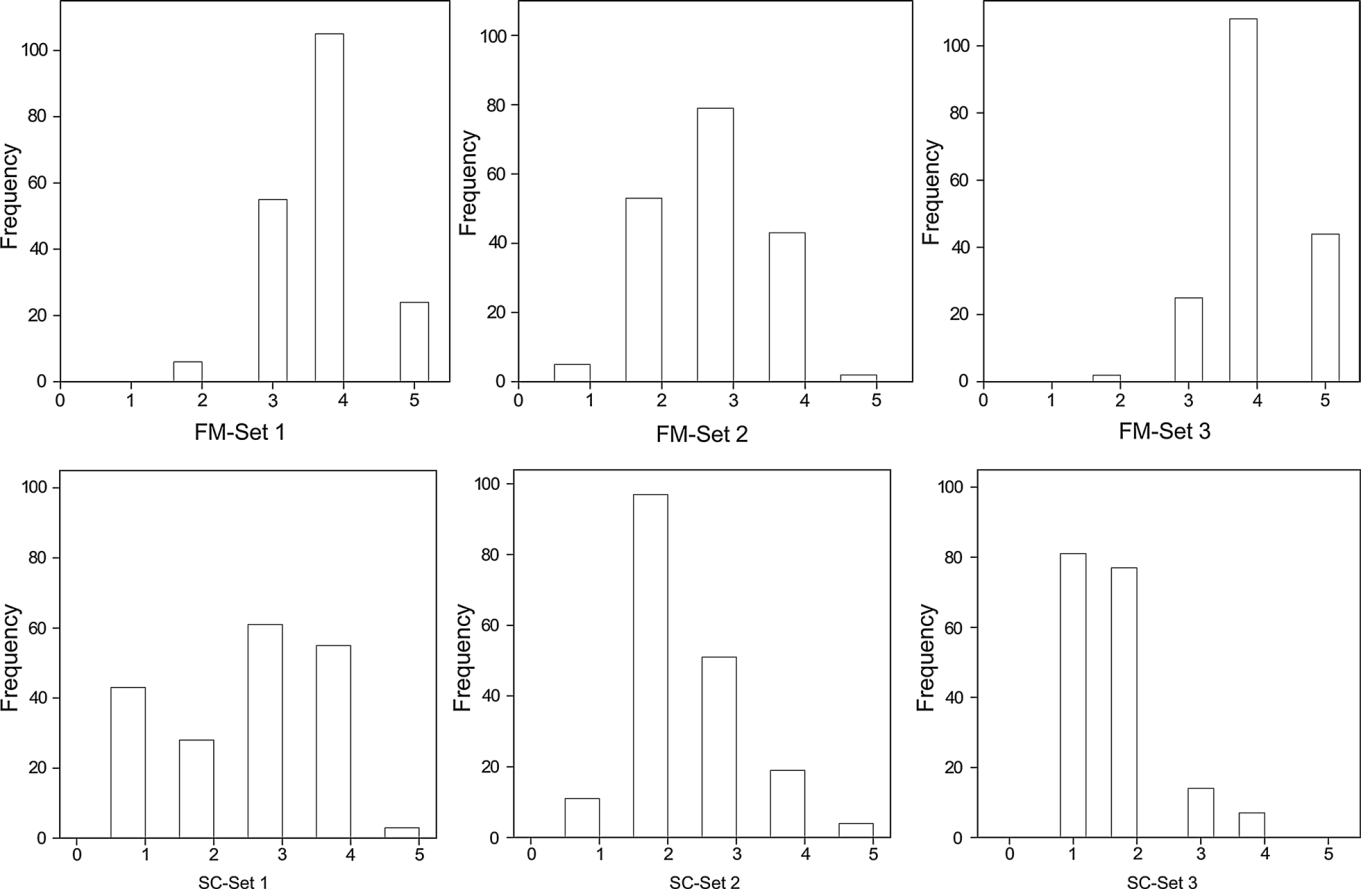
Frequency distribution histograms for traits FM and SC of heterokaryon Set 1, Set 2 and Set 3. The *horizontal axis* indicates data range of traits, and the *vertical axis* indicates the frequency of individuals

The frequency distribution of the three sets of heterokaryons showed continuous variation for almost all the traits (Figs. 2, 3). Cap color is controlled by one major locus on chromosome 8 and brown is partially dominant over white (Foulongne-Oriol et al. 2012a; Gao et al. 2015). Since the parental homokaryons used to generate Population 1 are white and brown, and both tester lines are white, approximately half of the individuals were white and the other half non-white (varying from white, cream, light brown to brown). Cap color shows, therefore, a bimodal distribution in these two sets of heterokaryons. Individuals of Set 2 and Set 3 colonized faster than those of Set 1. On day 14 after spawning, around 150 individuals of Set 2 and 170 individuals of Set 3 had 100 % COCO having the score of 10, while only 12 individuals of Set 1 had the score of 10. The continuous distribution of traits indicates quantitative features and polygenic control of these traits.

Genotype effect was significant (*α* = 0.05) for most traits apart from DS of Set 2 and SC of Set 3. Genetic background (tester line) was a significant factor to influence most traits except for CC and ER (*α* = 0.05). Broad-sense heritability was high for all traits of three sets of heterokaryons ranging from 0.66 for COCO across tester lines (COCO across Set 1 and Set 2) to 0.97 for CC of Set 2 (Additional file 1: Table S2), indicating that phenotypic variation was mostly determined by genotypic effects.

Spearman’s rank correlation coefficients (*r*) among the traits were calculated per set of heterokaryons (Additional file 1: Tables S3–S5). A consistent negative correlation was observed between ER and CC in Set 1 and 2. Since high values of ER represent late individuals and high values of CC represent dark individuals, brown individuals generally produced earlier than white individuals. COCO was also negatively correlated with ER in Set 1 indicating that a fast colonization of compost leads to earlier mushroom production in this set. There was no consistent correlation between COCO and the other traits in the Set 2 and 3. For all three sets of heterokaryons, ER was positively correlated with FM indicating that late individuals tended to produce firm mushrooms. Some late productions might lead to less mushrooms which may partially explain a better FM. Since heterokaryon Set 1 and 2 are the same set of homokaryotic progeny crossed with different tester lines, correlations for the same traits across Set 1 and 2 were also tested; significant correlations (*α* = 0.05) were also observed between other traits (sample size 167) including ER (r = 0.32), DS (r = 0.23) and SC (r = 0.22).

### QTL mapping of multiple traits

The multi-trait QTL model allows the analysis of several traits simultaneously, and thus the genetic basis of the correlations between traits. The covariance between traits was explained by the same QTLs having pleiotropic effects or QTLs being linked to the same chromosome. Because of the restricted recombination of *A. bisporus* var. *bisporus*, the estimated QTL positions are usually covering the whole linkage group, therefore QTLs are indicated according to the chromosome (CHR) number. Through multi-trait QTL analyses, the most significant QTL region of Set 1 was CHR10 with a significance of 28.3 for the −log_10_(*p*) (threshold = 2.7); the most significant QTL of Set 2 was CHR8, and the −log_10_(*p*) value was 9.6 (threshold = 2.7); CHR10 was also the most significant QTL of Set 3, and the −log_10_(*p*) value was 12.8 (threshold = 2.7). As expected, traits having phenotypic correlations mostly shared pleiotropic regions. Different QTLs were found in Set 1 and Set 2 indicating the influence of genetic background (tester lines).

### Cap color (CC)

Cap color was finally presented as a single trait due to its huge significance in multi-trait analyses (masking the results of the other traits), and a single QTL was detected on CHR8 in Set 1 and Set 2. The explained variance was 87.6 % for Set 1 and 86.4 % for Set 2 (Additional file 1: Table S6), which was in agreement with the major QTL of CC in the previous studies (Foulongne-Oriol et al. 2012a; Gao et al. 2015).

### Compost colonization (COCO)

**Fig. 4:**
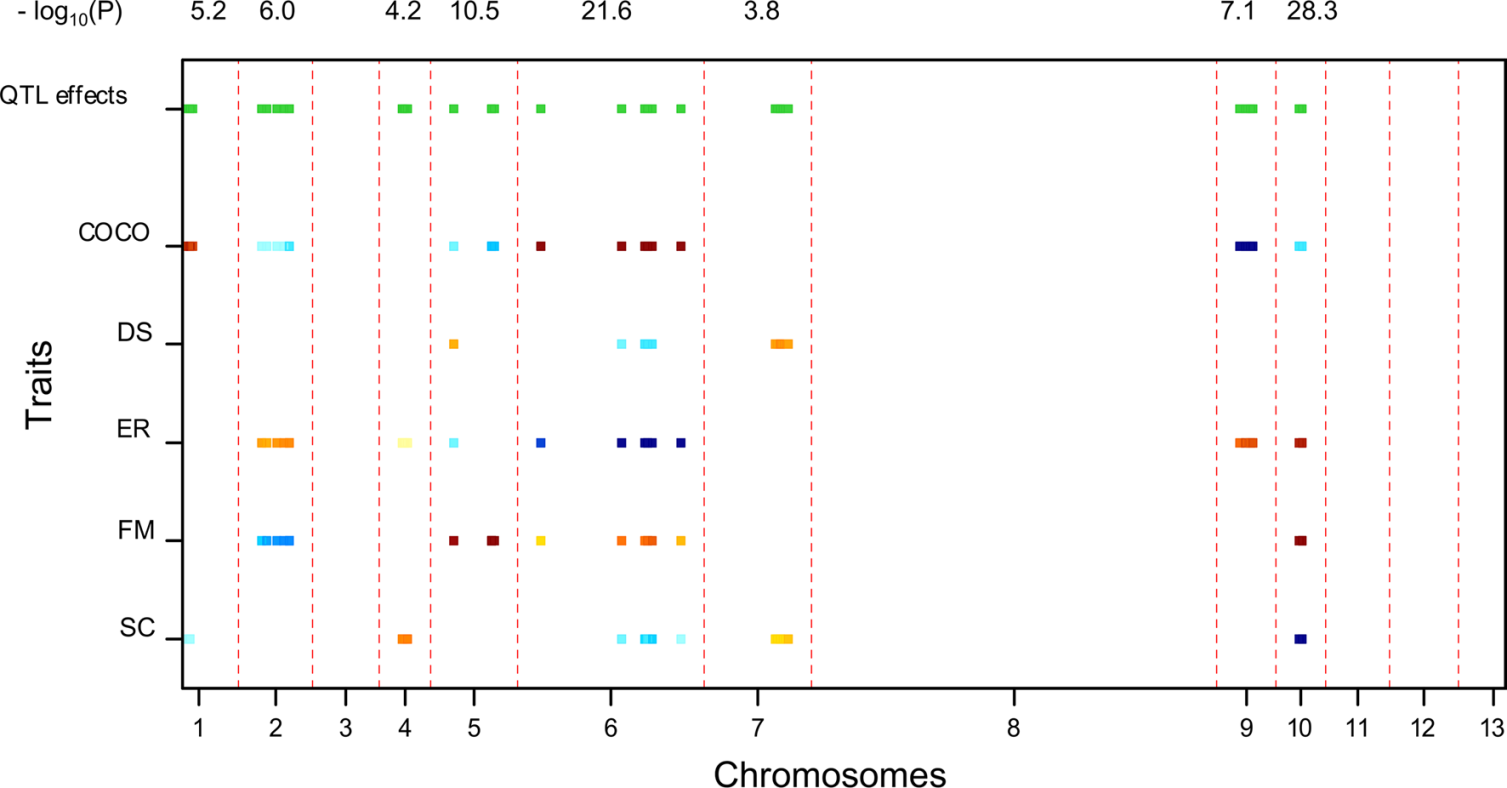
Multi-trait QTL analysis in heterokaryon Set 1. The two *color scales* of the *bars* indicate the two parents contributing high value allele of QTLs. The variation in *blue* (varying from *dark blue* to *light blue*) represents alleles from Mes09143 and the *darker* the color the higher the effect of the QTL. Similar for the variation in *red* (varying from *red* to *yellow*) that represents the high value alleles from H97. *Green bars* at the *top* indicate chromosomes bearing QTL with significant effects. The significance of the QTL were indicated above the figure in the value of “−log_10_(*p*)”. The different widths of linkage groups reflect the different map lengths of the corresponding chromosomes and represent thus the recombination frequency of markers on each chromosome. It shows that chromosome 8 has the highest frequency of recombination of all chromosomes

**Fig. 5:**
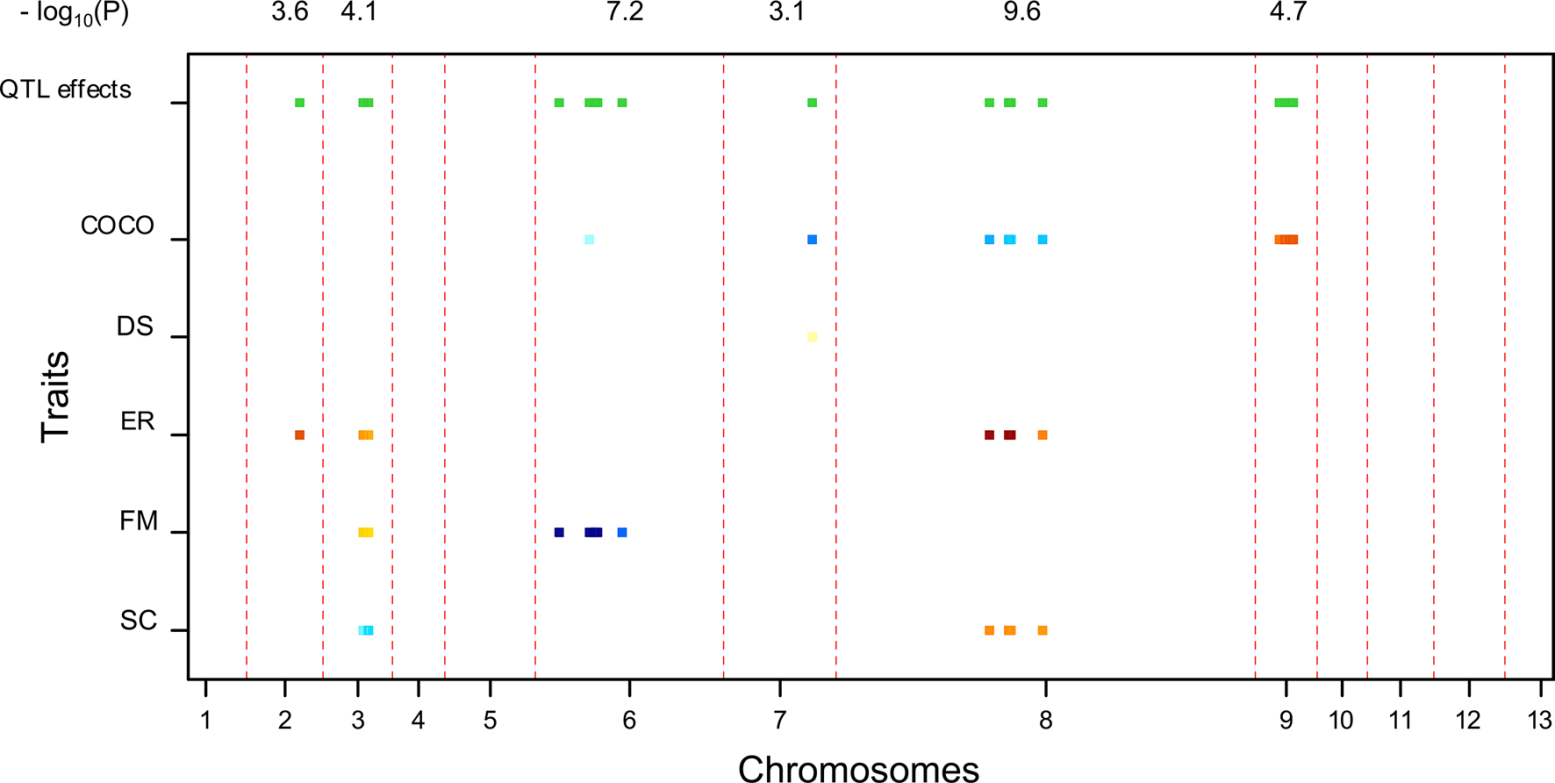
Multi-trait QTL analysis in heterokaryon Set 2. The two *color scales* of the *bars* indicate the two parents contributing high value alleles of QTLs. The variation in *blue* (varying from *dark blue* to *light blue*) represents alleles from Mes09143 and the *darker* the color the higher the effect of the QTL. Similar for the variation in *red* (varying from *red* to *yellow*) that represents the high value alleles from H97. *Green bars* at the *top* indicate chromosomes bearing QTL with significant effects. The significance of the QTL were indicated above the figure in the value of “−log_10_(*p*)”. The different widths of linkage groups reflect the different map lengths of the corresponding chromosomes and represent thus the recombination frequency of markers on each chromosome. It shows that chromosome 8 has the highest frequency of recombination of all chromosomes

**Fig. 6:**
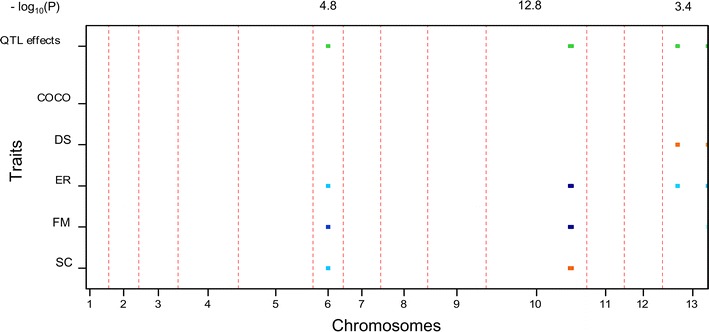
Multi-trait QTL analysis in heterokaryon Set 3. The two *color scales* of the *bars* indicate the two parents contributing high value alleles of QTLs. The variation in *blue* (varying from *dark blue* to *light blue*) represents alleles from H39 and the *darker* the color the higher the effect of the QTL. Similar for the variation in *red* (varying from *red* to *yellow*) that represents the high value alleles from Z8. *Green bars* at the *top* indicate chromosomes bearing QTL with significant effects. The significance of the QTL were indicated above the figure in the value of “−log_10_(*p*)”. The different widths of linkage groups reflect the different map lengths of the corresponding chromosomes and represent thus the recombination frequency of markers on each chromosome

Several QTLs were found for COCO in Set 1 and 2 (Figs. 4, 5), but none for Set 3 (Fig. 6). Consistent QTLs over Set 1 and 2 was detected on CHR6 and CHR9. CHR6 explained 16.5 % of the COCO variation for Set 1 (Table 3), while it only explained 2.4 % COCO variation for Set 2 (Table 4). The high value allele for CHR6 in Set 1 was from parent H97, and that of CHR6 in Set 2 was from the other parent Mes09143. The QTL CHR9 explained 10 and 8.4 % COCO variation for Set 1 and Set 2, respectively, while the high value allele was contributed by different parent, i.e., Mes09143 for Set 1 and H97 for Set 2 (Table 4). The different origin of the high value alleles for the same QTL region indicates the interaction effect of the genotype and tester lines.

**Table 3:** Explained variance (%) of QTLs detected in heterokaryon Set 1

LG	1	2	4	5	6	7	9	10
Locus name	ChrI_M1	ChrII_B1	ChrIV_B1	MHchrV_01	MHchrVI_02	MHchrVII_05	ChrIX_T1	ChrX_B1
Position	1.60	5.88	1.05	8.69	22.90	13.04	1.17	0.62
−log_10_(*p*)	5.242	6.043	4.176	10.451	21.562	3.789	7.130	28.253
Traits								
COCO	6.9^a^	1.6		3.4^a^	16.5^a^		10.0^a^	2.2
DS				2.2	3.7	5.5		
ER		3.9	2.7		15.4^a^		6.2^a^	9.6^a^
FM	1.5	4.7^a^	1.8	13.6^a^	6.4^a^			13.8^a^
SC	1.4		4.9^a^		2.5	3.0		31.4^a^

^a^QTLs that were also detected by single trait analyses. The others were not significant in the single trait analyses, while they are mostly just below the threshold of significance

**Table 4:** Explained variance (%) of QTLs detected in heterokaryon Set 2

LG	2	3	6	7	8	9
Locus name	MHchrII_05	ChrIII_B1	MHchrVI_01	MHchrVII_01	ChrVIII_B1	ChrIX_B3
Position	6.07	4.49	6.38	13.62	27.28	2.91
−log10(*p*)	3.584	4.086	7.231	3.075	9.649	4.690
Traits						
COCO			2.4	5.1^a^	3.9	8.4^a^
DS				3.0		
ER	8.4^a^	5.4^a^			13.9^a^	
FM		4.5^a^	12.2^a^		1.8	
SC		3.0			8.0 ^a^	

^a^QTLs that were also detected by single trait analyses. The others were not significant in the single trait analyses, while they are mostly just below the threshold of significance

### Distribution (DS)

QTLs of DS were mostly minor in all the three sets of heterokaryons. CHR13 showed the largest amount of explained variance in Set 3 (8.6 %) (Table 5). CHR7 was a consistent QTL over Set 1 and Set 2, which was not detected as a significant QTL with single trait analysis.

**Table 5:** Explained variance (%) of QTLs detected in heterokaryon Set 3

LG	6	10	13
Locus name	HZchrVI_03	CHR10A1	CHR13B1
Position	0	22.74	0
−log_10_(*p*)	4.771	12.717	3.393
Traits			
COCO			
DS			8.6^a^
ER	4.4^a^	14.2^a^	4.0 ^a^
FM	9.6^a^	14.9^a^	
SC	4.4	8.3^a^	

^a^QTLs that were also detected by single trait analyses. The others were not significant in the single trait analyses, while they are mostly just below the threshold of significance

### Earliness (ER)

Earliness of different sets of heterokaryons was controlled by different QTLs. For Set 1, two major QTLs for ER was detected on CHR6 and CHR10, explaining 15.4 and 9.6 % of the phenotypic variation; the ‘late’ allele (high ER value) was contributed by Mes09143 (CHR6) and H97 (CHR10), respectively (Fig. 4). It indicates CHR6 and CHR10 affecting ER on opposite directions. For Set 2, a major QTL for ER was on CHR8 explaining 13.9 % phenotypic variation, and here the allele associated with later production was from the late parent H97 (Fig. 5). QTL on CHR10 explaining 14.2 % of the phenotypic variation for Set 3, and the “late” allele was from H39 (Fig. 6). CHR2 was consistent for Set 1 and 2; CHR6 and CHR10 were consistent over Set 1 and Set 3. These QTLs were also detected with single trait analysis. (Additional file 1: Table S5).

### Firmness (FM)

CHR5 and CHR10 explained 13.6 and 13.8 % FM variation of Set 1, respectively. The high value alleles (firmer mushrooms) of the two QTLs were both from H97. CHR6 was a consistent QTL for the three heterokaryon sets, with explained variation of 6.4 % in Set 1, 12.2 % in Set 2 and 9.6 % in Set 3. However, the high value allele of FM in Set 1 was from H97, while that of FM in Set 2 was from Mes09143. It might suggest the interaction effect of genotype and tester lines. CHR10 explained larger phenotypic variation of FM (14.9 %) than CHR6 in Set 3, and the high value alleles of these two QTLs were both from the firmer parent H39.

### Scales (SC)

A major QTL CHR10 explained 31.4 % SC variation in Set 1 (Table 3), and the high value allele (smooth mushroom, less scaling) was indeed contributed by smoother parent Mes09143. CHR10 explained 8.0 % of SC variation in Set 3 (Table 5), and the high value alleles were from Z8. Only two minor QTLs (CHR3 and 8) were detected in Set 2 (Table 4), of which CHR3 was not significant in single trait analysis. The high value alleles of CHR8 was from H97. The origin of the high value alleles corresponded to the fact that Horst U1 shows more scaling than WB2 and WW7.

## Discussion

Most of the present-day button mushroom varieties are genetically very similar (Moore et al. 2001; Sonnenberg et al. 2011). They are all susceptible to the same diseases, have the same biological efficiency on substrates and have very similar quality traits. New varieties with improved traits are only commercially viable if they meet at least the quality standards of the present varieties. Previous and present breeding programs have shown that restoration of quality and agronomic traits while introducing new traits by outcrossing with wild germplasm is very difficult. One of the main reasons is the extraordinary recombination landscape in the variety *bisporus* causing substantial linkage drag. Almost all the crossover events locate at the very tips of chromosome ends. These crossovers are often missed in segregation analysis, since covering chromosome ends with suitable markers is difficult for the presence of telomeres and associated repeats (Sonnenberg et al. 2016). Previous researchers thus concluded that recombination is rare in the variety *bisporus* compared to the *burnettii* variety, whereas the main differences between these varieties lies in the position of crossovers, i.e., at chromosome ends for the *bisporus* variety and a more even distributed over chromosomes for the *burnettii* variety. As a result, introgression breeding is hampered by substantial linkage drag, and QTL mapping is limited to the assignment of QTLs to chromosomes. Nevertheless, the lack of knowledge on the genetic basis of most agronomic and quality traits is a bottleneck for breeding and our research can contribute to its understanding. Especially, the multi-trait QTL analysis will help to understand the interdependency of traits.

Significant correlations indicate that traits referring to agronomic features and quality of button mushrooms might be interrelated and influence each other (Additional file 1: Tables S3–S5). Previous research has shown that ER correlated with yield (Foulongne-Oriol et al. 2012a) and disease resistance (Foulongne-Oriol et al. 2011). In this study, we found that ER of mushroom production associated with CC, i.e., brown individuals generally produced earlier than white individuals. It is unlikely that CC is directly influencing the time of production, and it is more likely that genomic regions involved in ER are linked to regions determining CC for the brown lines used here. In heterokaryon Set 2, QTLs for CC and ER were located on the same chromosome (CHR8). However, due to the absence of crossovers in the major part of each chromosome it is unclear that if these QTLs represent pleiotropic QTL having effects on multiple traits or closely linked genes. Agronomic and quality traits are complex and not only determined by genetic factors but also determined by environmental factors. The expression of correlated traits may also be modified by the same environmental factors.

In cultivation trials we used the maximum number of genotypes (instead of less genotypes in replicates) in order to generate as much genetic variation as possible. Each individual (genotype) was thus grown once in one box, which may relatively weaken the statistical power of the analyses. Nevertheless, we actually reproduce the analysis in a different way. Heterokaryon Set 1 and Set 2 were generated by one segregating population crossed with two different tester lines. Different tester lines represent different genetic backgrounds of the same segregating population, and the two heterokaryon sets were cultivated in two independent cultivation trials. Although they were cultivated once only, we actually reproduce to score the segregating population twice under different genetic backgrounds, rather than technically reproduce the experiment by multiple cultivations. The QTL we identified for CC in heterokaryon Set 1 and Set 2 positively proved the reproducibility of the data. Major QTLs for CC of Set 1 and Set 2 were both detected on chromosome 8 with 87.6 % explained variation for Set 1 and 86.4 % of that for Set 2. This results were consistent with that found in previous studies (Foulongne-Oriol et al. 2012a; Gao et al. 2015). Since quality-related traits, especially morphology traits change over time during mushroom development, we scored all traits at developmental stage 3 (Hammond and Nichols 1976). Mushroom development is a complex and dynamic process, and environmental conditions can also change in time. Thus, these traits could also be assessed on multiple time points during the cropping cycle and represented as a function of time. A model for the study of the genetic architecture of complex and dynamic traits, so called functional mapping has been generated previously (Lin and Wu 2006). Data of time series have been incorporated into the growth model and QTL analysis in potato (Hurtado et al. 2012). Here we excluded data of flush 2 since the environment for each individual in flush 2 is probably quite different, such as the amount of nutrients available in the substrate, pH and water potential in compost and casing soil.

Since good crop varieties combine optimal values for several traits to maximize productivity and quality, multiple traits of germplasm should be evaluated during a breeding scheme rather than single traits. The simplest approach to analyse multiple traits is to perform a series of single-trait analyses and then combine the results. However, a combined multi-trait analysis can be more powerful than a collection of single-trait analyses (Malosetti et al. 2008). In this study the major QTLs for all traits were detected in both the single- and the multi-trait QTL analyses. It seems that more consistent minor QTLs were detected in the multi-trait analyses although one has to be aware that the statistical assumptions are stronger for the multi-trait analysis, which may cause more false positive than single trait analysis. QTLs detected by single-trait analyses were mostly detected also in multi-trait analysis except for four minor ones (Additional file 1: Table S6). All QTL-detected chromosomes represent two or more traits. CHR6 and CHR10 represents the highest number of traits, and they are thus important chromosomes for breeding of quality and agronomic traits. Most chromosomes harbour QTLs in our study. Similarly, QTLs of productivity and quality in *Pleurotus ostreatus* appear to be scattered across the genome and were shown to have small effects on the variation of the corresponding traits (Larraya et al. 2003).

Cap color is an important quality trait for traders and consumers. The consumer preference seems to be for either pure white or dark brown. White cultivars represent still the largest part of the market, but the sale of brown button mushrooms is gradually increasing (Callac et al. 1998). Cap color is a quantitative trait controlled by a major locus on CHR8 and several minor loci (Foulongne-Oriol et al. 2012a; Gao et al. 2015). Since CC is mainly determined by one locus resulting in a white or non-white phenotype QTL mapping of this trait leads to extremely high LOD value and was, therefore, not included in the multi-trait analysis. Single-trait QTL analysis confirmed the presence of the major QTL on chromosome 8 explaining up to 87.6 % of the phenotypic variation in CC (Additional file 1: Table S6), which is in agreement with that reported in the previous studies. Cap color was measured with CIELAB image system in those two studies. In our study, the color scoring ranged from 1 to 5, and that might explain the absence of the minor QTLs. The minor QTL for CC, however, might represent important modifier genes that influence the color intensity. A shorter crop cycle is relevant for the production costs. Production ER is positively correlated with the speed of COCO, i.e., the faster the COCO, the earlier the mushroom production. Five QTLs for ER were detected previously on chromosome 1, 2, 5, 10 and 11 in an intervarietal segregating population (Foulongne-Oriol et al. 2012a). Three of these were also detected in this study, but not the ones on CHR1 and CHR11. Over all QTLs detected for ER, the beneficial alleles for earlier mushrooms were mostly contributed by the wild parents (Mes09143 or Z8). If these QTLs can be assigned to smaller chromosome regions, it may facilitate marker-assisted breeding for an earlier producing cultivar. Smoothness of mushroom cap is an important quality trait, because SC on mushroom cap discolor easily post-harvest and reduce thus the quality. Although the two parental heterokaryons of Set 1 and Set 3 were not significantly different in scaling, the high value alleles beneficial for smoothness of the major QTL (31.4 % explained variance) were from the wild parent Mes09143 and Z8, which might be useful for breeding smoother mushroom caps.

CHR10 and CHR6 have effects on most traits. CHR10 was identified as a major QTL for resistance of *Lecanicillium fungicola* (dry-bubble disease) (Foulongne-Oriol et al. 2012b) and for bruising sensitivity in button mushroom (Gao et al. 2015). Earliness were found highly correlated with disease incidence, and the earliest strains appeared to be the most resistant ones (Foulongne-Oriol et al. 2011). Earliness and disease resistance might be genetically correlated and controlled by linked or shared genetic factors. Nevertheless, the association between the two traits was probably affected by common environmental factors. Mycelia of button mushrooms and disease usually compete with each other during COCO. Earlier individuals colonize the compost faster than late individuals thereby leaving less time and fewer opportunities for disease to start the infection. In this case, disease could be considerably minimized by using earlier mushroom cultivars which have fast growing and fruiting ahead of time.

As shown in Results, QTL locations and the parental origin of the high value alleles have more overlap for Set 1 and Set 3. Since Set 1 and 2 represent the same set of homokaryotic offspring one would expect a better overlap in QTL between Set 1 and Set 2. This apparent inconsistency can be explained well by examining the genetic relationship between these two sets of heterokaryons (Fig. 1). Heterokaryon Set 1 contains alleles in one nucleus from either H97 (A) or Mes09143 (C) and in the other nucleus only alleles from the tester H39 (B). Set 3 contains alleles in one nucleus from H39 (B) or Z8 (F) and alleles in the other nucleus from the tester H97 (A). Both sets of heterokaryons share thus, on average, 75 % of the alleles derived from Horst U1 (A B). In both sets of heterokaryons alleles from the original Horst U1 (H39 × H97) are thus combined and indeed most good traits present in Horst U1 map to the relevant parent in these populations.

Here we made a start at understanding the genetic base for a number of agronomic and quality traits in button mushrooms. This was done using for the first time in mushroom breeding a multi-trait QTL analysis. Although the experimental set up was limited, this approach shows that QTL can be found in this way and that it also elucidates relationships between traits. Due to the extraordinary recombination landscape of the bisporic variety (Sonnenberg et al. 2016), mapping for precise QTL locations is not possible, and it is difficult to say if the QTLs identified on the same chromosomes represent the same genes with pleiotropic effects or closely linked genes. Previous research has indicated that recombination frequency in the tetra-sporic variety *burnettii* (Foulongne-Oriol et al. 2010) is relatively normal. Identifying genes involved in this important trait would allow the introduction of recombination in the bisporic variety. Since the two varieties are fully compatible and the trait is dominant, this seems to be feasible. That would allow a more precise mapping and reduction of linkage drag. In addition, with the development of the next-generation sequencing and the availability of the abundant wild mushroom germplasm, genome wide association study (GWAS) might be an alternative way to identify SNPs closely linked to genes controlling interesting agronomic traits.

## Additional file

10.1186/s13568-016-0239-3 Supplementary tables.

## Abbreviations

ER: earliness
FM: firmness
CC: cap color
COCO: compost colonization
SC: scales
DS: mushroom distribution
CHR: chromosome
QTL: quantitative trait locus
QTLs: quantitative trait loci
BIC: Bayesian information criterion
CIM: composite interval mapping
H^2^: broad-sense heritability
