# Supplementary material for: Multi-trait QTL analysis for agronomic and quality characters of Agaricus bisporus (button mushrooms)
Source: AMB Express. 2016 Sep 8;6(1):67. doi: 10.1186/s13568-016-0239-3 (PMC5016490; doi:10.1186/s13568-016-0239-3)
Supplement: Supplementary file 1 — 10.1186/s13568-016-0239-3 Supplementary tables. [file 13568_2016_239_MOESM1_ESM.pdf]

Supplementary Materials

AMB Express

Multi-trait QTL analysis for agronomic and quality characters of *Agaricus bisporus* (button mushrooms)

Wei Gao[1, 2*], Johan JP Baars[1], Chris Maliepaard[1], Richard GF Visser[1], Jinxia Zhang[1], Anton SM Sonnenberg[1]

[1]*Wageningen UR Plant Breeding, Wageningen University & Research Centre, 6700AA Wageningen, The Netherlands*

[2]*Institute of Agricultural Resources and Regional Planning, Chinese Academy of Agricultural Sciences, 100081 Beijing, PR China*

[*]Corresponding author.

Fax: +86-10-82106207

Tel: +86-10-82108761

E-mail address: gaowei01@caas.cn (Wei Gao)

Table S1. Information of strains used in this study

Function	Ploidy	Source	Strain name	Number in the collection
Parental line	Heterokaryon		Horst U1	MES02989
	Heterokaryon		WB2	MES01535
	Heterokaryon		WW7	MES01808
	homokaryon	Protoplastal clone	H97	MES12135
	homokaryon	Protoplastal clone	H39	MES12146
	homokaryon	Protoplastal clone	Mes09143	MES09143
	homokaryon	Protoplastal clone	Z6	MES13581
	homokaryon	Protoplastal clone	Z8	MES13573
Population 1	homokaryon	Single spore isolation (SSI)	F1H04	F1H04
	homokaryon	SSI	F1H08	F1H08
	homokaryon	SSI	F1H15	F1H15
	homokaryon	SSI	F1H42	F1H42
	homokaryon	SSI	F1H47	F1H47
	homokaryon	SSI	F1H59	F1H59
	homokaryon	SSI	F1H60	F1H60
	homokaryon	SSI	F1H63	F1H63
	homokaryon	SSI	F1H64	F1H64
	homokaryon	SSI	F1H71	F1H71
	homokaryon	SSI	F1H73	F1H73
	homokaryon	SSI	F1H80	F1H80
	homokaryon	SSI	F1H81	F1H81
	homokaryon	SSI	F1H85	F1H85
	homokaryon	SSI	F1L01	F1L01
	homokaryon	SSI	F1L02	F1L02
	homokaryon	SSI	F1L03	F1L03
	homokaryon	SSI	F1L04	F1L04
	homokaryon	SSI	F1L13	F1L13
	homokaryon	SSI	F1L17	F1L17
	homokaryon	SSI	F1L29	F1L29
	homokaryon	SSI	F1L33	F1L33
	homokaryon	SSI	F1L38	F1L38
	homokaryon	SSI	F1L40	F1L40
	homokaryon	SSI	F1O03	F1O03
	homokaryon	SSI	F1O05	F1O05
	homokaryon	SSI	F1O18	F1O18
	homokaryon	SSI	F1O20	F1O20
	homokaryon	SSI	F1O22	F1O22
	homokaryon	SSI	F1O25	F1O25

homokaryon	SSI	F1O38	F1O38
homokaryon	SSI	F1O40	F1O40
homokaryon	SSI	F1O51	F1O51
homokaryon	SSI	F1O64	F1O64
homokaryon	SSI	F1O65	F1O65
homokaryon	SSI	F2H02	F2H02
homokaryon	SSI	F2H17	F2H17
homokaryon	SSI	F2H18	F2H18
homokaryon	SSI	F2H20	F2H20
homokaryon	SSI	F2H21	F2H21
homokaryon	SSI	F2H28	F2H28
homokaryon	SSI	F2H30	F2H30
homokaryon	SSI	F2H34	F2H34
homokaryon	SSI	F2H52	F2H52
homokaryon	SSI	F2H57	F2H57
homokaryon	SSI	F2H59	F2H59
homokaryon	SSI	F2H61	F2H61
homokaryon	SSI	F2H62	F2H62
homokaryon	SSI	F2H64	F2H64
homokaryon	SSI	F2H66	F2H66
homokaryon	SSI	F2L111	F2L111
homokaryon	SSI	F2L29	F2L29
homokaryon	SSI	F2L54	F2L54
homokaryon	SSI	F2L59	F2L59
homokaryon	SSI	F2L89	F2L89
homokaryon	SSI	F2L92	F2L92
homokaryon	SSI	F2L93	F2L93
homokaryon	SSI	F2L95	F2L95
homokaryon	SSI	F2L96	F2L96
homokaryon	SSI	F2L99	F2L99
homokaryon	SSI	F2O10	F2O10
homokaryon	SSI	F2O12	F2O12
homokaryon	SSI	F2O14	F2O14
homokaryon	SSI	F2O28	F2O28
homokaryon	SSI	F2O29	F2O29
homokaryon	SSI	F2O32	F2O32
homokaryon	SSI	F2O34	F2O34
homokaryon	SSI	F2O42	F2O42
homokaryon	SSI	F2O54	F2O54
homokaryon	SSI	F2O61	F2O61
homokaryon	SSI	MH02	MH02
homokaryon	SSI	MH03	MH03
homokaryon	SSI	MH111	MH111
homokaryon	SSI	MH115	MH115

homokaryon	SSI	MH121	MH121
homokaryon	SSI	MH122	MH122
homokaryon	SSI	MH124	MH124
homokaryon	SSI	MH129	MH129
homokaryon	SSI	MH13	MH13
homokaryon	SSI	MH133	MH133
homokaryon	SSI	MH135	MH135
homokaryon	SSI	MH136	MH136
homokaryon	SSI	MH137	MH137
homokaryon	SSI	MH138	MH138
homokaryon	SSI	MH14	MH14
homokaryon	SSI	MH141	MH141
homokaryon	SSI	MH143	MH143
homokaryon	SSI	MH15	MH15
homokaryon	SSI	MH158	MH158
homokaryon	SSI	MH160	MH160
homokaryon	SSI	MH163	MH163
homokaryon	SSI	MH181	MH181
homokaryon	SSI	MH185	MH185
homokaryon	SSI	MH187	MH187
homokaryon	SSI	MH193	MH193
homokaryon	SSI	MH196	MH196
homokaryon	SSI	MH197	MH197
homokaryon	SSI	MH199	MH199
homokaryon	SSI	MH208	MH208
homokaryon	SSI	MH215	MH215
homokaryon	SSI	MH216	MH216
homokaryon	SSI	MH217	MH217
homokaryon	SSI	MH218	MH218
homokaryon	SSI	MH22	MH22
homokaryon	SSI	MH224	MH224
homokaryon	SSI	MH23	MH23
homokaryon	SSI	MH242	MH242
homokaryon	SSI	MH248	MH248
homokaryon	SSI	MH26	MH26
homokaryon	SSI	MH262	MH262
homokaryon	SSI	MH263	MH263
homokaryon	SSI	MH264	MH264
homokaryon	SSI	MH271	MH271
homokaryon	SSI	MH276	MH276
homokaryon	SSI	MH277	MH277
homokaryon	SSI	MH279	MH279
homokaryon	SSI	MH281	MH281
homokaryon	SSI	MH282	MH282

homokaryon	SSI	MH284	MH284
homokaryon	SSI	MH287	MH287
homokaryon	SSI	MH304	MH304
homokaryon	SSI	MH305	MH305
homokaryon	SSI	MH309	MH309
homokaryon	SSI	MH318	MH318
homokaryon	SSI	MH32	MH32
homokaryon	SSI	MH321	MH321
homokaryon	SSI	MH327	MH327
homokaryon	SSI	MH34	MH34
homokaryon	SSI	MH348	MH348
homokaryon	SSI	MH352	MH352
homokaryon	SSI	MH356	MH356
homokaryon	SSI	MH358	MH358
homokaryon	SSI	MH361	MH361
homokaryon	SSI	MH365	MH365
homokaryon	SSI	MH366	MH366
homokaryon	SSI	MH367	MH367
homokaryon	SSI	MH372	MH372
homokaryon	SSI	MH374	MH374
homokaryon	SSI	MH382	MH382
homokaryon	SSI	MH386	MH386
homokaryon	SSI	MH390	MH390
homokaryon	SSI	MH413	MH413
homokaryon	SSI	MH425	MH425
homokaryon	SSI	MH442	MH442
homokaryon	SSI	MH453	MH453
homokaryon	SSI	MH459	MH459
homokaryon	SSI	MH46	MH46
homokaryon	SSI	MH461	MH461
homokaryon	SSI	MH463	MH463
homokaryon	SSI	MH464	MH464
homokaryon	SSI	MH469	MH469
homokaryon	SSI	MH474	MH474
homokaryon	SSI	MH483	MH483
homokaryon	SSI	MH49	MH49
homokaryon	SSI	MH497	MH497
homokaryon	SSI	MH500	MH500
homokaryon	SSI	MH501	MH501
homokaryon	SSI	MH508	MH508
homokaryon	SSI	MH512	MH512
homokaryon	SSI	MH517	MH517
homokaryon	SSI	MH524	MH524
homokaryon	SSI	MH526	MH526

	homokaryon	SSI	MH540	MH540
	homokaryon	SSI	MH542	MH542
	homokaryon	SSI	MH548	MH548
	homokaryon	SSI	MH551	MH551
	homokaryon	SSI	MH59	MH59
	homokaryon	SSI	MH60	MH60
	homokaryon	SSI	MH64	MH64
	homokaryon	SSI	MH65	MH65
	homokaryon	SSI	MH68	MH68
	homokaryon	SSI	MH70	MH70
	homokaryon	SSI	MH71	MH71
	homokaryon	SSI	MH77	MH77
	homokaryon	SSI	MH80	MH80
	homokaryon	SSI	MH83	MH83
	homokaryon	SSI	MH93	MH93
	homokaryon	SSI	MH94	MH94
	homokaryon	SSI	MH99	MH99
	homokaryon	SSI	T104	T104
	homokaryon	SSI	T123	T123
	homokaryon	SSI	T127	T127
	homokaryon	SSI	T132	T132
	homokaryon	SSI	T135	T135
	homokaryon	SSI	T175	T175
	homokaryon	SSI	T184	T184
	homokaryon	SSI	T193	T193
	homokaryon	SSI	T200	T200
	homokaryon	SSI	T209	T209
	homokaryon	SSI	T211	T211
	homokaryon	SSI	T221	T221
Population 2	homokaryon	SSI	F1A05	F1A05
	homokaryon	SSI	F1A22	F1A22
	homokaryon	SSI	F1A28	F1A28
	homokaryon	SSI	F1A29	F1A29
	homokaryon	SSI	F1A34	F1A34
	homokaryon	SSI	F1A36	F1A36
	homokaryon	SSI	F1A40	F1A40
	homokaryon	SSI	F1A43	F1A43
	homokaryon	SSI	F1B04	F1B04
	homokaryon	SSI	F1B06	F1B06
	homokaryon	SSI	F1B20	F1B20
	homokaryon	SSI	F1B50	F1B50
	homokaryon	SSI	F1C103	F1C103
	homokaryon	SSI	F1C106	F1C106

homokaryon	SSI	F1C110	F1C110
homokaryon	SSI	F1C125	F1C125
homokaryon	SSI	F1C133	F1C133
homokaryon	SSI	F1C14	F1C14
homokaryon	SSI	F1C140	F1C140
homokaryon	SSI	F1C46	F1C46
homokaryon	SSI	F1C61	F1C61
homokaryon	SSI	F1C74	F1C74
homokaryon	SSI	F1C75	F1C75
homokaryon	SSI	F1C77	F1C77
homokaryon	SSI	F1C84	F1C84
homokaryon	SSI	F2A01	F2A01
homokaryon	SSI	F2A02	F2A02
homokaryon	SSI	F2A03	F2A03
homokaryon	SSI	F2A16	F2A16
homokaryon	SSI	F2A33	F2A33
homokaryon	SSI	F2B61	F2B61
homokaryon	SSI	F2B68	F2B68
homokaryon	SSI	F2B70	F2B70
homokaryon	SSI	F2C23	F2C23
homokaryon	SSI	F2C26	F2C26
homokaryon	SSI	F2C41	F2C41
homokaryon	SSI	F2C46	F2C46
homokaryon	SSI	F2C52	F2C52
homokaryon	SSI	F2C76	F2C76
homokaryon	SSI	HZ116	HZ116
homokaryon	SSI	HZ117	HZ117
homokaryon	SSI	HZ122	HZ122
homokaryon	SSI	HZ124	HZ124
homokaryon	SSI	HZ127	HZ127
homokaryon	SSI	HZ129	HZ129
homokaryon	SSI	HZ131	HZ131
homokaryon	SSI	HZ133	HZ133
homokaryon	SSI	HZ136	HZ136
homokaryon	SSI	HZ16	HZ16
homokaryon	SSI	HZ17	HZ17
homokaryon	SSI	HZ170	HZ170
homokaryon	SSI	HZ173	HZ173
homokaryon	SSI	HZ174	HZ174
homokaryon	SSI	HZ182	HZ182
homokaryon	SSI	HZ198	HZ198
homokaryon	SSI	HZ21	HZ21
homokaryon	SSI	HZ23	HZ23
homokaryon	SSI	HZ231	HZ231

homokaryon	SSI	HZ234	HZ234
homokaryon	SSI	HZ25	HZ25
homokaryon	SSI	HZ258	HZ258
homokaryon	SSI	HZ261	HZ261
homokaryon	SSI	HZ264	HZ264
homokaryon	SSI	HZ268	HZ268
homokaryon	SSI	HZ27	HZ27
homokaryon	SSI	HZ273	HZ273
homokaryon	SSI	HZ274	HZ274
homokaryon	SSI	HZ290	HZ290
homokaryon	SSI	HZ292	HZ292
homokaryon	SSI	HZ295	HZ295
homokaryon	SSI	HZ298	HZ298
homokaryon	SSI	HZ300	HZ300
homokaryon	SSI	HZ303	HZ303
homokaryon	SSI	HZ319	HZ319
homokaryon	SSI	HZ32	HZ32
homokaryon	SSI	HZ320	HZ320
homokaryon	SSI	HZ33	HZ33
homokaryon	SSI	HZ333	HZ333
homokaryon	SSI	HZ34	HZ34
homokaryon	SSI	HZ340	HZ340
homokaryon	SSI	HZ341	HZ341
homokaryon	SSI	HZ343	HZ343
homokaryon	SSI	HZ346	HZ346
homokaryon	SSI	HZ360	HZ360
homokaryon	SSI	HZ371	HZ371
homokaryon	SSI	HZ372	HZ372
homokaryon	SSI	HZ373	HZ373
homokaryon	SSI	HZ376	HZ376
homokaryon	SSI	HZ379	HZ379
homokaryon	SSI	HZ382	HZ382
homokaryon	SSI	HZ387	HZ387
homokaryon	SSI	HZ389	HZ389
homokaryon	SSI	HZ39	HZ39
homokaryon	SSI	HZ392	HZ392
homokaryon	SSI	HZ398	HZ398
homokaryon	SSI	HZ400	HZ400
homokaryon	SSI	HZ406	HZ406
homokaryon	SSI	HZ412	HZ412
homokaryon	SSI	HZ418	HZ418
homokaryon	SSI	HZ425	HZ425
homokaryon	SSI	HZ427	HZ427
homokaryon	SSI	HZ43	HZ43

homokaryon	SSI	HZ431	HZ431
homokaryon	SSI	HZ440	HZ440
homokaryon	SSI	HZ442	HZ442
homokaryon	SSI	HZ45	HZ45
homokaryon	SSI	HZ458	HZ458
homokaryon	SSI	HZ460	HZ460
homokaryon	SSI	HZ466	HZ466
homokaryon	SSI	HZ468	HZ468
homokaryon	SSI	HZ477	HZ477
homokaryon	SSI	HZ485	HZ485
homokaryon	SSI	HZ487	HZ487
homokaryon	SSI	HZ49	HZ49
homokaryon	SSI	HZ493	HZ493
homokaryon	SSI	HZ501	HZ501
homokaryon	SSI	HZ502	HZ502
homokaryon	SSI	HZ511	HZ511
homokaryon	SSI	HZ514	HZ514
homokaryon	SSI	HZ519	HZ519
homokaryon	SSI	HZ520	HZ520
homokaryon	SSI	HZ522	HZ522
homokaryon	SSI	HZ532	HZ532
homokaryon	SSI	HZ537	HZ537
homokaryon	SSI	HZ538	HZ538
homokaryon	SSI	HZ54	HZ54
homokaryon	SSI	HZ540	HZ540
homokaryon	SSI	HZ542	HZ542
homokaryon	SSI	HZ545	HZ545
homokaryon	SSI	HZ572	HZ572
homokaryon	SSI	HZ575	HZ575
homokaryon	SSI	HZ576	HZ576
homokaryon	SSI	HZ577	HZ577
homokaryon	SSI	HZ580	HZ580
homokaryon	SSI	HZ584	HZ584
homokaryon	SSI	HZ598	HZ598
homokaryon	SSI	HZ599	HZ599
homokaryon	SSI	HZ60	HZ60
homokaryon	SSI	HZ602	HZ602
homokaryon	SSI	HZ608	HZ608
homokaryon	SSI	HZ61	HZ61
homokaryon	SSI	HZ63	HZ63
homokaryon	SSI	HZ630	HZ630
homokaryon	SSI	HZ635	HZ635
homokaryon	SSI	HZ636	HZ636
homokaryon	SSI	HZ637	HZ637

homokaryon	SSI	HZ642	HZ642
homokaryon	SSI	HZ676	HZ676
homokaryon	SSI	HZ683	HZ683
homokaryon	SSI	HZ692	HZ692
homokaryon	SSI	HZ693	HZ693
homokaryon	SSI	HZ696	HZ696
homokaryon	SSI	HZ703	HZ703
homokaryon	SSI	HZ704	HZ704
homokaryon	SSI	HZ716	HZ716
homokaryon	SSI	HZ723	HZ723
homokaryon	SSI	HZ74	HZ74
homokaryon	SSI	HZ743	HZ743
homokaryon	SSI	HZ749	HZ749
homokaryon	SSI	HZ754	HZ754
homokaryon	SSI	HZ758	HZ758
homokaryon	SSI	HZ761	HZ761
homokaryon	SSI	HZ765	HZ765
homokaryon	SSI	HZ77	HZ77
homokaryon	SSI	HZ778	HZ778
homokaryon	SSI	HZ788	HZ788
homokaryon	SSI	HZ794	HZ794
homokaryon	SSI	HZ800	HZ800
homokaryon	SSI	HZ811	HZ811
homokaryon	SSI	HZ85	HZ85
homokaryon	SSI	HZ851	HZ851
homokaryon	SSI	HZ853-2	HZ853-2
homokaryon	SSI	HZ87	HZ87
homokaryon	SSI	HZ870	HZ870
homokaryon	SSI	HZ888	HZ888
homokaryon	SSI	HZ889	HZ889
homokaryon	SSI	HZ894	HZ894
homokaryon	SSI	HZ918	HZ918
homokaryon	SSI	HZ921	HZ921
homokaryon	SSI	HZ98	HZ98

Table S2. Broad-sense Heritability of traits in different heterokaryotic populations

Heterokaryon Set and Population	Trait	σ^2_G	σ^2_e	H^2
Heterokaryon Set 1	CC	1.79	0.12	0.97
Population 1 (crossed with tester line H39)	DS	1.06	0.71	0.75
Data across flushes	FM	0.81	0.36	0.82
	SC	1.25	0.80	0.76
Heterokaryon Set 2	CC	2.29	0.12	0.98
Population 1 (crossed with tester line Z6)	DS	0.77	0.63	0.71

Data across flushes	FM	0.88	0.53	0.77
	SC	0.74	0.55	0.73
Population 1 (Heterokaryon Set 1 and Set 2)	COCO	2.48	2.60	0.66
Data across tester lines	DS	0.97	0.65	0.75
	ER	3.11	1.48	0.81
	FM	0.62	0.55	0.69
	SC	1.27	0.84	0.75
Heterokayron Set 3	DS	1.15	0.66	0.78
Population 2 (crossed with tester line H97)	FM	0.85	0.29	0.86
Data across flushes	MT	0.57	0.31	0.79
	SC	0.73	0.67	0.69

Table S3. Correlation coefficients among traits of heterokaryon set 1 (n=177)

	CC	COCO	DS	ER	FM	SC
CC	*					
COCO	-	*				
DS	-	-	*			
ER	-0.22	-0.43	-	*		
FM	-0.20	-	-	-	*	
SC	-	-	-	-	-0.35	*

Spearman’s rank correlation between traits of heterokaryon set 1. “-” indicates a non-significant correlation coefficient. All listed Spearman correlation coefficients are significant *(α*=0.05).

Table S4. Correlation coefficients among traits of heterokaryon set 2 (n=185)

	CC	COCO	DS	ER	FM	SC
CC	*					
COCO	0.16	*				
DS	-	-	*			
ER	-0.35	-	-	*		
FM	-	-	-0.16	0.30	*	
SC	-0.26	-	-	-	-	*

Spearman’s rank correlation between traits of heterokaryon set 3. “-” indicates a non-significant correlation coefficient. All listed Spearman correlation coefficients are significant (α=0.05).

Table S5. Correlation coefficients among traits of heterokaryon set 3 (n=180)

	COCO	DS	ER	FM	SC
COCO	*				
DS	-	*			
ER	-	-	*		
FM	-	-	0.49	*	
SC	-	-	-	-	*

“-” indicates non-significant correlation. All listed Spearman correlation coefficients are significant (α=0.05).

Table S6. QTLs of all traits detected by single trait QTL analysis

Heterokaryon set	Locus no.	Locus name	Linkage group	Position	_LOG10(P)	%Expl. Var.	Add. eff.	High value allele	s.e.
CC/Set 1	95	MHchrVIII_03	8	29.5	81.699	87.576	0.938	Mes09143	0.028
CC/Set 2	95	MHchrVIII_03	8	29.5	76.561	86.431	0.932	Mes09143	0.029
COCO/Set 1	2	ChrI_M1	1	1.6	4.895	7.128	0.268	H97	0.06
	45	MHchrV_01	5	8.69	2.858	3.598	0.19	Mes09143	0.059
	59	MHchrVI_02	6	22.9	10.483	16.747	0.41	H97	0.058
	125	ChrIX_T1	9	1.17	5.999	8.556	0.293	Mes09143	0.058
COCO/Set 2	66	MHchrVII_01	7	0	3.86	6.795	0.261	Mes09143	0.067
	126	MHchrIX_03	9	2.23	4.763	8.332	0.289	H97	0.066
	153	C13P2	13	1.98	5.034	9.358	0.307	H97	0.067
ER/Set 1	59	MHchrVI_02	6	22.9	8.922	15.933	0.4	Mes09143	0.062
	125	ChrIX_T1	9	1.17	4.405	7.145	0.268	H97	0.064
	131	ChrX_B1	10	0.62	5.159	8.906	0.299	H97	0.065
ER/Set 2	11	C2P2	2	2.15	5.693	10.082	0.318	H97	0.065
	18	MHchrIII_05	3	1.08	5.522	9.238	0.305	H97	0.063
	34	ChrV_T1	5	0	4.157	6.68	0.259	Mes09143	0.064
	95	MHchrVIII_03	8	29.5	7.744	14.233	0.378	H97	0.064
ER/Set 3	16	HZchrIII_05	3	0	3.95	5.797	0.241	H39	0.061
	24	HZchrIV_06	4	0	4.981	7.952	0.283	H39	0.062
	48	HZchrVI_02	6	0	3.152	4.711	0.218	H39	0.063
	59	C8P2	8	1.59	5.021	8.08	0.285	Z8	0.062
	90	CHR10A1	10	22.74	9.763	17.992	0.425	H39	0.063
	115	*C13P6	13	5.72	3.847	6.964	0.265	H39	0.068
DS/Set 1	57	C6P21	6	20.7	2.884	5.397	0.233	Mes09143	0.071
	77	*MHchrVII_03	*7	11.37	3.379	6.374	0.253	H97	0.07
DS/Set 2	125	ChrIX_T1	9	1.17	2.976	5.767	0.241	H97	0.072
DS/Set 3	113	*C13P3	13	2.86	3.643	8.642	0.295	Z8	0.078
FM/Set 1	13	MHchrII_04	2	4.65	4.182	5.695	0.239	Mes09143	0.059
	38	C5P7	5	6.51	6.587	10.436	0.324	H97	0.061
	61	MHchrVI_01	6	23.96	4.161	5.671	0.239	H97	0.059
	131	ChrX_B1	10	0.62	9.362	15.135	0.39	H97	0.059
	152	*MHchrXIII_05	*13	0	3.716	5.079	0.226	Mes09143	0.059

FM/Set 2	18	MHchrIII_05	3	1.08	3.231	5.599	0.237	H97	0.068
	62	C6P26	6	25.5	6.077	12.839	0.359	Mes09143	0.07
FM/Set 3	48	HZchrVI_02	6	0	5.019	10.426	0.324	H39	0.071
	90	CHR10A1	10	22.74	6.801	13.984	0.375	H39	0.069
SC/Set 1	32	ChrIV_B1	4	1.05	5.024	7.663	0.278	H97	0.061
	131	ChrX_B1	10	0.62	13.833	26.629	0.517	Mes09143	0.062
SC/Set 2	94	C8P28	8	27.79	3.249	6.69	0.259	H97	0.074
SC/Set 3	87	C10P18	10	18.19	5.15	12.845	0.359	Z8	0.078

"*" indicates QTLs detected only in single trait analysis but not in multi-trait analysis.
